# Sesquiterpene Lactones: Promising Natural Compounds to Fight Inflammation

**DOI:** 10.3390/pharmaceutics13070991

**Published:** 2021-06-30

**Authors:** Melanie S. Matos, José D. Anastácio, Cláudia Nunes dos Santos

**Affiliations:** 1 Instituto de Biologia Experimental e Tecnológica (iBET), Apartado 12, 2781-901 Oeiras, Portugal; melanie.matos@ibet.pt (M.S.M.); jose.diogo.anastacio@nms.unl.pt (J.D.A.); 2 Instituto de Tecnologia Química e Biológica António Xavier, Universidade Nova de Lisboa, Av. da República, 2780-157 Oeiras, Portugal; 3 CEDOC, Chronic Diseases Research Centre, NOVA Medical School, Faculdade de Ciências Médicas, Universidade NOVA de Lisboa, Campo dos Mártires da Pátria, 130, 1169-056 Lisboa, Portugal

**Keywords:** anti-inflammatory, bioactivity, sesquiterpene lactone-rich natural extracts, germacranolides, guaianolides, eudesmanolides, heliangolides, pseudoguainolides, inflammatory pathway, NF-κB, pro-inflammatory mediators

## Abstract

Inflammation is a crucial and complex process that reestablishes the physiological state after a noxious stimulus. In pathological conditions the inflammatory state may persist, leading to chronic inflammation and causing tissue damage. Sesquiterpene lactones (SLs) are composed of a large and diverse group of highly bioactive plant secondary metabolites, characterized by a 15-carbon backbone structure. In recent years, the interest in SLs has risen due to their vast array of biological activities beneficial for human health. The anti-inflammatory potential of these compounds results from their ability to target and inhibit various key pro-inflammatory molecules enrolled in diverse inflammatory pathways, and prevent or reduce the inflammatory damage on tissues. Research on the anti-inflammatory mechanisms of SLs has thrived over the last years, and numerous compounds from diverse plants have been studied, using in silico, in vitro, and in vivo assays. Besides their anti-inflammatory potential, their cytotoxicity, structure–activity relationships, and pharmacokinetics have been investigated. This review aims to gather the most relevant results and insights concerning the anti-inflammatory potential of SL-rich extracts and pure SLs, focusing on their effects in different inflammatory pathways and on different molecular players.

## 1. Sesquiterpene Lactones

Sesquiterpene lactones (SLs) are composed of a large and diverse group of phytochemicals found in numerous plant families, with the greatest number of compounds belonging to the *Asteraceae* family [1]. These molecules derive from two main precursors, isopentenyl diphosphate (IPP), and dimethylallyl diphosphate (DMAPP) [2,3]. These precursors can be generated in plants via either the mevalonate pathway, which occurs within the cytosol, or the 2-C-methyl-D-erythritol pathway, occurring in the chloroplasts [2,3]. IPP and DMAPP are converted into farnesyl diphosphate (FPP) by the enzyme farnesyl diphosphate synthase [4,5]. FPP is considered a common precursor for SLs, but can be further converted to sterols, triterpenes, or used for prenylationed of proteins. Its cyclization by germacrene A synthase into germacrene A is considered as the first step towards SL production [2]. Germacrene A is subsequently oxidized by a cytochrome P450 enzyme, germacrene A oxidase, into germacrene A acid. This molecule can be further modified to produce different subclasses of SLs, with the characteristic 15-carbon backbone structure [4,5,6, 7].

**Figure 1:**
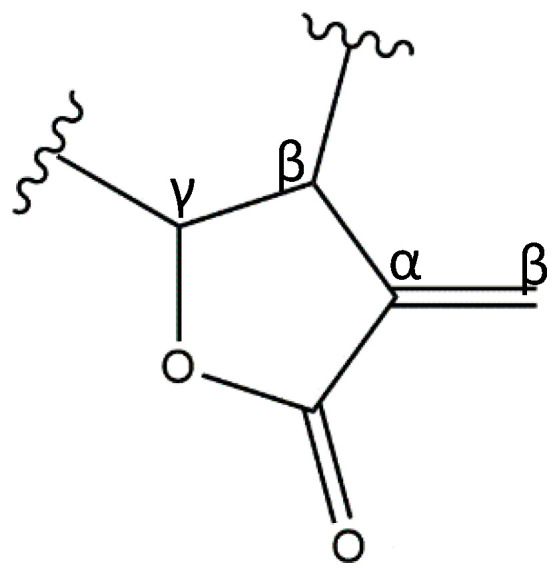
α-methylene-γ-lactone moiety core structure characteristic of SLs.

**Figure 2:**
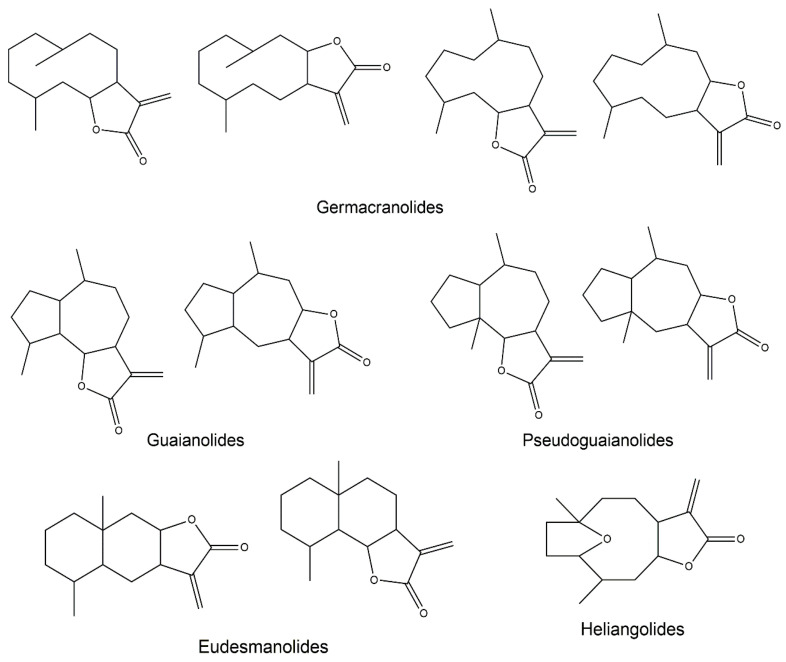
Structural backbone of the main SL subclasses.

SLs are characterized by a 15-carbon backbone containing α,β-unsaturated carbonyl structures, such as a conserved α-methylene-γ-lactone (Figure 1). Numerous other modifications to the molecular structure are also known, including the presence of hydroxyls, esters, and epoxides [8,9]. There are over 5000 reported structures of SLs, divided into several skeletal subtypes, the main ones being germacranolides, guaianolides, eudesmanolides, heliangolides, and pseudoguaianolides (Figure 2) [1,10].

In recent years, the interest in SLs has risen considerably due to their vast array of biological activities relevant to human health. This curiosity has led to many studies regarding the isolation of these compounds from natural sources, the development of complete or semi-synthesis procedures, and the evaluation of the pharmacological potential of SLs and their derivatives. Some of the many known activities reported for this class of compounds include anti-microbial, anti-fungal, anti-viral, anti-tumor, anti-malarial, anti-diabetic, analgesic, and anti-inflammatory activities [1,11]. There is a close relationship between the pharmacological potential of SLs and their respective ecological purposes, since their main goals in nature consist of anti-microbial and anti-herbivore endeavors, as well as the growth inhibition of competing plants [12].

**Figure 3:**
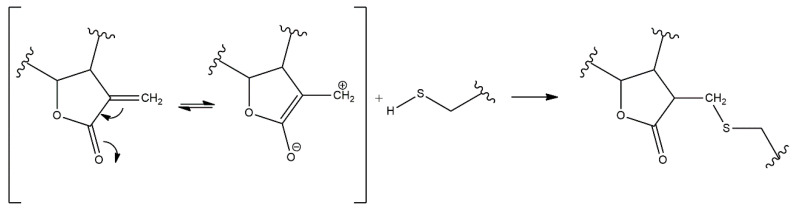
Michael reaction between an α-methylene-γ-lactone moiety and a sulfhydryl group.

The electrophilic α,β-unsaturated carbonyl moieties present in SLs, which react by Michael-type addition with biological nucleophiles, can explain the wide range of observed biological activities. In particular, sulfhydryl groups present in biomolecules, such as glutathione (GSH) or proteins with free cysteine groups, are major targets for alkylation by SLs (Figure 3) [8,10]. Consequently, the function of these biomolecules may become significantly impaired and lead to decreased activity in the case of enzymes, or disruption of the cellular redox balance through interference with the GSH metabolism, which ultimately promotes apoptosis [1]. Although covalent modifications are considered the lead mechanism of action carried out by most SLs, non-covalent interactions with specific molecules can also play a part in the bioactivity of these compounds [10].

Although the effects of SLs depend on varied factors, namely, the compound structure, the concentration used, and the organism assayed, SLs are generally considered highly toxic compounds, due to their somewhat unspecific mechanism of action. In fact, structural features responsible for therapeutic bioactivities are sometimes the ones responsible for toxic effects. Nonetheless, given their pronounced biological potential and “drug-like” physicochemical properties (based on Lipinski’s rule of five), SLs have been looked upon as very promising lead compounds.

## 2. Anti-Inflammatory Potential of Sesquiterpene Lactones

### 2.1. General Notes on Inflammation

Inflammation is a complex immune response that restores tissue homeostasis after a noxious stimulus, such as infection or tissue injury. It is a crucial process but can become detrimental when it occurs in excess due to its tissue-damaging potential since several types of unspecific effector molecules are released, causing collateral damage. In the case of transitory abnormal conditions, an acute inflammatory response successfully restores the system’s basal homeostatic setpoints after pathogen clearance or tissue repair. However, if the inflammatory trigger persists, the ongoing inflammatory state shifts the system to different setpoints, and acute inflammation may progress to chronic inflammation and, ultimately, disease [13,14]. Chronic inflammation is involved in ailments such as asthma, allergies, rheumatoid arthritis, multiple sclerosis, psoriasis, or inflammatory bowel diseases, and is known to be associated with a higher risk of cancer development [15].

Glucocorticoids and nonsteroidal anti-inflammatory drugs (NSAIDs) are the most common anti-inflammatory drugs. However, they present several adverse side effects after prolonged exposure. Thereby, the identification of novel compounds that can resolve inflammation without the adverse side effects caused by currently used anti-inflammatory therapies is of the utmost importance.

The different pathways by which SLs present their anti-inflammatory potential will be further discussed in the following sections of this review. Indeed, research on the anti-inflammatory mechanisms of action of pure SLs and SL-rich extracts has thrived over the last few years, with a vast number of publications resorting to in silico, in vitro, and in vivo models. This review aims to gather the most relevant results and insights concerning the anti-inflammatory potential of SL-containing extracts and pure SLs, focusing on their effect on different inflammatory pathways.

### 2.2. SL-Containing Extracts

The rise of SLs as promising bioactive molecules is a consequence of the use of SL-rich plant extracts in traditional medicine for the treatment of various ailments over the centuries. Reports of the anti-inflammatory potential of natural extracts containing SLs can be found in many studies dedicated to different plant species from the *Asteraceae* family, which is characterized by having structurally diverse SLs [16]. Both in vitro and in vivo evidence indicates that this class of compounds can exert their effect on several inflammatory pathways (Table 1).

A chicory root (*Cichorium intybus* L.) extract, rich in dihydrolactucin, lactucin, deoxylactucin, jacquinelin, and dihydrolactucopicrin, dose-dependently downregulated the gene expression of inducible cyclooxygenase (COX-2) (IC_50_ = 117 μg/mL), inducible nitric oxide synthase (iNOS) (IC_50_ = 39 μg/mL), as well as the pro-inflammatory cytokines tumor necrosis factor-alpha (TNF-α) (IC_50_ = 48 μg/mL) and interleukin-1-beta (IL-1β) (IC_50_ = 22 μg/mL) in lipopolysaccharide (LPS)-stimulated RAW264.7 macrophages, while no inhibitory effect on the expression of constitutive COX-1 was observed [17]. In the same study, to elucidate which compounds were responsible for the extract activity, the authors isolated the main SLs from the chicory extract, and the guaianolides 8-deoxylactucin and dihydrolactucopicrin were revealed as the most active compounds [17]. Similar effects were observed using the same in vitro model (LPS-stimulated macrophages) in the case of an *Artemisia leucodes* L. extract enriched in the guaianolides austricin and leukomisin [18]. These two pure compounds were also tested individually, and it was demonstrated that neither of them alone could explain the decreased gene expression of COX-2 and iNOS observed for the extract. This suggests that the overall effect of the extract probably results from the combined effects of each of the components therein, possibly with synergistic interactions [18]. In these two studies, SL-containing extracts presented anti-inflammatory effects comparable to those of known drugs. That is the case with oral administration of the chicory extract, which demonstrated a comparable effect to that of indomethacin in a carrageenan-induced rat paw edema model, by reducing the inflammation and paw volume [17]. The same extract displayed a prolonged effect in a collagen-induced arthritis model, by significantly reducing inflammation until 5 days after the end of the treatment [17]. In the case of *Artemisia leucodes*, oral administration of the extract itself was more effective than aspirin in reducing the swelling in a rat paw edema model, as well as reducing the immune cell infiltrate and granuloma formation in a cotton granuloma test (chronic inflammation challenge) [18]. This underlines that, in some cases, an extract containing several compounds may produce a more potent anti-inflammatory response than one pure compound.

Similarly, the treatment of LPS-induced J774A.1 macrophages with an SL-rich fraction from *Artemisia khorassanica* L. reduced nitric oxide (NO), TNF-α, IL-1β, as well as prostaglandin E_2_ (PGE_2_), which is one of the main products of the arachidonic acid (AA) cascade resulting from COX activity [19]. In this case, the nuclear factor kappa-light-chain-enhancer of activated B cells (NF-κB) activity was also reduced, which could justify these effects, since many pro-inflammatory genes including those coding for COX-2, iNOS, and several pro-inflammatory cytokines (such as TNF-α and IL-1β) display a binding site for NF-κB in their promoter region [18,19]. In another study that focused on the anti-inflammatory bioactivity of *Artemisia* extracts in J774A.1 macrophages, the SL composition of several *Artemisia* species was evaluated by proton nuclear magnetic resonance (^1^H-NMR) experiments [20]. The authors concluded that *Artemisia* species enriched in saturated SLs seemed to be more potent inhibitors of NO and PGE_2_ production than those richer in unsaturated SL structures [20].

A dichloromethane extract from *Eupatorium perfoliatum* L. containing SLs was tested in RAW264.7 macrophages, and it was able to reduce both iNOS expression and NO production. The main compounds identified in the extract were isolated and investigated, revealing that the dimeric guaianolide diguaiaperfolin and the flavonoid eupafolin were the main active constituents. The extract also decreased the expression of several cytokines at both the gene and protein levels, namely the IL-1 and TNF families, as well as IL-6, which is generally produced as a response to stimulation by the previous two, and the colony-stimulating factor-3 (CSF-3), responsible for activating granulocytes. The authors suggested that the effect caused by the extract could have been in part due to eupafolin, a non-SL compound identified as one of the main active constituents of the extract [21].

*Xanthium spinosum* L. methanolic extract inhibited COX-1 and 12-lipoxygenase (12-LOX) enzymatic pathways in human platelets and increased the synthesis of the anti-inflammatory eicosanoid 15-Hydroxyeicosatetraenoic acid (15(S)-HETE), which is an inhibitor of phospholipase A_2_ [22]. The extract also inhibited 5-lipoxygenase (5-LOX) in rat polymorphonuclear leukocytes (PMNLs), and a 5-LOX bioguided fractionation of the extract resulted in the isolation of the known 12,8-guaianolide ziniolide [22]. A 1 h pre-incubation with either the extract or isolated ziniolide was capable of inhibiting NF-κB signaling after a 7 h inflammatory stimulus, which may contribute to a more lasting anti-inflammatory effect. However, the more immediate inhibitory effects on eicosanoid biosynthesis, observed after a short incubation of only a few minutes, resulted from the direct interaction with the AA pathway enzymes rather than the mediation by NF-κB inhibition [22].

Extracts prepared from the Arbo and Spanish varieties of *Arnica montana* L., rich in helenalin and dihydrohelenalin esters, inhibited IL-1β and TNF-α release in peripheral blood mononuclear cells (PBMCs), as well as the deoxyribonucleic acid (DNA)-binding of the transcription factors NF-κB and the nuclear factor of activated T-cells (NFAT) in Jurkat cells, both of which are responsible for the transcription of pro-inflammatory genes [23]. The Arbo variety was shown to be more effective than the Spanish one, a result attributed to the fact that the main SLs present in the former were helenalin-derivatives, as opposed to the predominant dihydrohelenalin-derivatives in the latter [23]. These results underline the importance of the α-methylene-γ-lactone moiety in the bioactive potential of SLs. Besides the core structure of the SL, it was demonstrated in Jurkat T cells that the SL derivatives esterified with unsaturated acids, such as methacrylate or tiglinate, were more active than those esterified with saturated acids, such as acetate, a result that was confirmed in vivo when 11α,13-dihydrohelenalin methacrylate was shown to be more effective than 11α,13-dihydrohelenalin acetate in inhibiting the swelling in a mouse ear edema model [23]. It is also worth mentioning that preliminary studies carried out by the authors suggested that the mechanism of NFAT inhibition is different from the one described for the known immunosuppressants tacrolimus (FK506) and cyclosporin [23], which highlights the potential of SLs as alternative anti-inflammatory leads to circumvent the side effects of currently used drugs.

In a study comprising extracts from different *Centaurea* species obtained with different solvents (*n*-hexane, chloroform, or methanol), chloroform extracts were the most effective in inhibiting NF-κB and iNOS, bioactivities that were attributed to the presence of SLs, which tend to be preferentially extracted by this solvent due to their hydrophobicity [24]. A chloroform extract from *C. athoa* was highlighted as the one with the most promising anti-inflammatory potential. The extract inhibited NF-κB activity in vitro in human chondrosarcoma cells, to the same extent as the pure germacranolide parthenolide, which was considered as the positive control [24]. Moreover, oral administration of this same extract to rats reduced the swelling in an in vivo paw edema model [24]. A follow-up study from the same group revealed athoin, 14-*O*-acetylathoin, and methyl-14-*O*-acetylathoin-12-oate to be the main SLs present in *C. athoa* [25].

Gao et al. [26] suggested the oral use of the *Inula helenium* L. extract, mainly composed of alantolactone and isoalantolactone, for the prevention and treatment of rheumatoid arthritis, after the promising results obtained in vitro and in vivo. In particular, the extract inhibited NF-κB and MAPKs activation in bEnd.3 mouse endothelial cells, and decreased the release of the pro-inflammatory mediator IL-1, the monocyte chemoattractant protein (MCP-1), and matrix metalloproteinase (MMP)-3 in primary synovial fibroblasts from patients, as well as IL-6 and iNOS in murine macrophages [26]. Additionally, in rats, oral administration of the extract improved rheumatoid arthritis symptoms in both the developing and the developed phases of the disease [26].

Oral administration of a fraction from *Arctium lappa* L. enriched in the germacranolide onopordopicrin decreased colitis-associated histological damage in rats and prevented mucin layer loss, which is a common feature of inflammatory bowel diseases (IBD) responsible for a defective barrier function. Additionally, neutrophil infiltration was reduced, as well as the production of TNF-α and COX-2, whereas COX-1 was not affected [27].

The concentrations upon which SL molecules are pharmacologically active are not well defined, and although SLs are described as poorly bioavailable [28], the aforementioned in vivo reports show that, in many cases, orally administered SLs are pharmacologically active against inflammation. These conclusions highlight the anti-inflammatory potential of orally administered SLs, thereby reinforcing the importance of studying their pharmacokinetic (ADME—Absorption, Distribution, Metabolism, Excretion) profile of these molecules. On the other hand, a topical application was also shown to be effective in both acute and chronic inflammatory processes when a *Vernonia scorpioides* L. extract, containing diacethylpiptocarphol and related hirsutinolides, was used to treat dermatitis and psoriasis in mice [29]. The extract reduced edema, neutrophil infiltration, and epidermal hyperproliferation, possibly through the inhibition of the chemotactic cytokine IL-8 and NF-κB activity, and its effectiveness was comparable to that of dexamethasone [29].

Although many natural extracts containing SLs show promising anti-inflammatory potential (Table 1), one must keep in mind that extracts may be complex mixtures containing several compounds from different classes that might interact with each other producing synergistic or antagonistic effects. Therefore, while extracts may be useful as adjuvant therapies, the major potential is provided by individual compounds that might be considered leads for the pharmaceutical industry. For this reason, to validate their anti-inflammatory applicability, SLs must be isolated from their natural sources and their effect must be studied further. In the following section, the relevant studies based on pure SLs are gathered and are divided by SL subclasses.

### 2.3. Germacranolides

#### 2.3.1. Parthenolide

**Figure 4:**
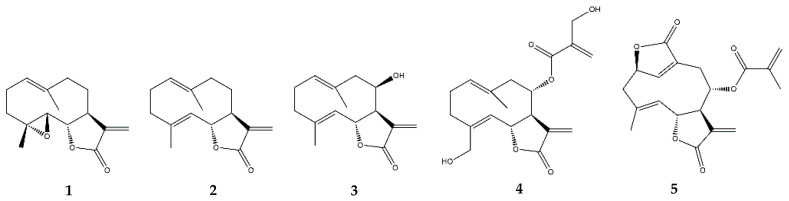
Structures of addressed germacranolides. **1**—Parthenolide; **2**—Costunolide; **3**—Eupatolide; **4**—Onopordopicrin; **5**—Deoxyelepantopin.

Germacranolides are undoubtedly the most extensively studied class of SLs due to the well-known pharmacological potential of parthenolide. It was first isolated from the medicinal plant feverfew (*Tanacetum parthenium* L.), which inspired the scientific community to further explore other germacranolides. Parthenolide (Figure 4) works as an anti-inflammatory and anti-cancer agent, and both bioactivities are mainly driven by its capacity to interfere with the NF-κB pathway, since this transcription factor controls the proliferative, the anti-apoptotic, as well as the pro-inflammatory genes [30]. Given that several signaling pathways rely on NF-κB, the anti-inflammatory success of parthenolide can be assessed by its effect on numerous indirect targets [30]. As an example, the treatment of LPS/interferon (IFN)-γ-stimulated rat aortic smooth muscle cells with parthenolide prevented NO release and iNOS gene expression, via the stabilization of the IκBα/NF-κB complex by inhibiting the degradation of the former and the nuclear translocation of the latter [31].

Parthenolide appears to have the potential to reduce brain inflammation, and its lipophilic character favors blood–brain barrier (BBB) permeability, factors that make this germacranolide a promising agent to treat glioblastomas and offer explanations for the anti-migraine efficacy of orally administered *Tanacetum parthenium* L., a major known source of parthenolide [32,33]. For instance, parthenolide dose-dependently decreased IL-6 and TNF-α release in LPS-stimulated BV-2 microglia [32], as well as rat primary neuro-glial cells [33], probably because of its strong ability to reduce NF-κB nuclear translocation and the subsequent transcription of pro-inflammatory genes. In rats, an injection of a low dosage of parthenolide before LPS stimulation attenuates oxidative stress, brain inflammation, and fever [33]. These effects could be explained by the decreased activation of NF-κB and nuclear respiratory factor (Nrf)-1 (a reactive oxygen species (ROS)-induced transcription factor), and by the reduced expression of COX-2 (critical in the generation of the known fever mediator PGE_2_) in the hypothalamus [33].

In another research study, parthenolide inhibited IL-4, and to a lesser extent, IL-2 and IFN-γ expression in Jurkat T cells activated by phorbol 12-myristate 13-acetate (PMA)/ionomycin or anti-CD3/CD28 antibodies, by blocking the NF-κB pathway. These results were confirmed in primary human T lymphocytes from both healthy and allergic donors when 2.5 µM parthenolide could completely suppress the IL-4 protein levels, while higher doses were required for the same effect to be achieved for IL-2 and IFN-γ. Although all three cytokines are NF-κB-regulated genes, their different responses to parthenolide raises the possibility of using this compound to treat allergic diseases, which are mainly mediated by the preferential differentiation of Th2 over Th1 lymphocytes, directed by IL-4 stimulation [34]. In another study, parthenolide inhibited the activation of T-cells in whole blood by hindering the expression of IL-2, a mediator of lymphocyte proliferation and activation [35]. In this case, the SL acted in a step prior to the differentiation into CD4 or CD8 T-cells, possibly by interfering with the activation of either the activator protein 1 (AP-1), NF-κB, NFAT or octamer transcription factor-1 (Oct-1), all of which are transcription factors that control IL-2 expression [35].

Li et al. [36] hypothesized that the inhibition of the Toll-like receptor 4 (TLR4) signaling pathway could reduce LPS-induced inflammation, since the binding of LPS to this receptor leads to MAPKs activation and the nuclear translocation of NF-κB to induce pro-inflammatory cytokine release. Indeed, parthenolide was able to inhibit the phosphorylation of MAPKs, IκBα, and p65, and counteract the upregulation of several pro-inflammatory cytokines, after LPS stimulation in monocytes. Because these inflammatory events can reflect TLR4 activation, and since parthenolide inhibits the LPS-induced upregulation of TLR4, the authors stated that the anti-inflammatory effects of this SL are due to its ability to inhibit the TLR4 signaling pathway. Nevertheless, although parthenolide may operate partly through TLR4-mediated signaling, its direct effect on the MAPK and NF-κB pathways must not be overlooked.

In a rat model, the intraperitoneal administration of either parthenolide or enhydrin, another germacranolide, significantly attenuated the paw edema and hyperalgesia caused by a carrageenan injection [37]. Such results make these germacranolides potential alternatives to NSAIDs. However, the SL derivative 11β,13-dihydroparthenolide was not able to reverse carrageenan-induced inflammatory nociception, possibly due to the lack of the α-methylene-γ-lactone group [37].

#### 2.3.2. Costunolide

**Figure 5:**
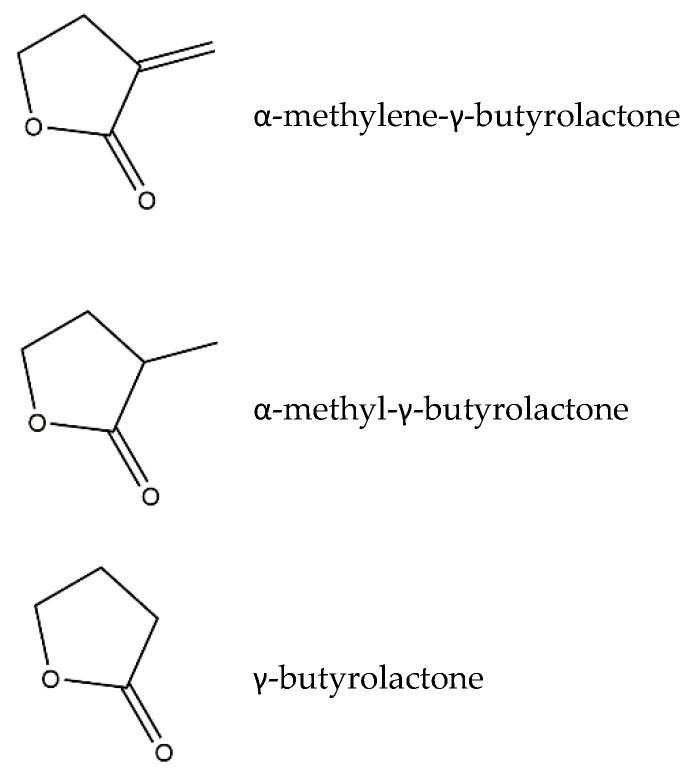
Structures of α-methylene-γ-butyrolactone, α-methyl-γ-butyrolactone, and γ-butyrolactone.

Costunolide (Figure 4), another well-described germacranolide, was first isolated from *Saussurea lappa* L. and can modulate several intracellular signaling pathways involved in inflammation. Costunolide’s mode of action is in many ways similar to that of parthenolide. In particular, the timing of the treatment for either parthenolide or costunolide to obtain an anti-inflammatory effect was proven to be critical, given that both SLs were more effective when applied before the inflammatory stimuli in a pre-treatment approach, rather than in a simultaneous incubation with the inflammatory insult [33,38]. Specifically, parthenolide can diminish an LPS-induced fever, inflammation, and oxidative stress in rats when injected before LPS stimulation but not when administered simultaneously with LPS [33]. In turn, costunolide was more effective in preventing IL-6 and TNF-α release in murine macrophages when the cells were pre-incubated with the SL before being elicited with LPS, as opposed to what was seen in the case of a simultaneous incubation with both costunolide and LPS [38]. These observations make sense, since the bioactivity of SLs is driven by a modulation of the transcription factors’ signaling pathways, which needs some time to occur. In addition, the importance of the presence of an α-methylene-γ-lactone group in costunolide also became evident when an α-methylene-γ-butyrolactone structure induced heme oxygenase-1 (HO-1) expression as well as Nrf-2 activity and nuclear translocation to the same extent as costunolide in RAW264.7 macrophages, while the α-methyl-γ-butyrolactone and γ-butyrolactone structures did not (Figure 5) [38]. The induction of the antioxidant enzyme HO-1 via the Nrf-2 pathway explained the ability of costunolide to decrease TNF-α and IL-6 [38].

Although the NF-κB pathway is one of the main targets of parthenolide and costunolide, other transcription factors have also been addressed as targets. In HepG2 hepatocytes, parthenolide inhibited the IL-6-induced expression of acute-phase protein genes, which are intimately related to inflammatory responses [39]. Parthenolide blocks the phosphorylation of signal transducers and the activators of transcription proteins (STATs), in particular STAT3, probably through the inactivation of the Janus kinases (JAKs) by conjugation with their thiol groups. Consequently, the STAT3 dimerization required for the nuclear translocation of the transcription complex does not take place [39]. Butturini et al. [40] drew similar conclusions concerning costunolide when it prevented the IL-6-elicited activation and the DNA-binding of STAT3 in the human monocytic cell line THP-1. These observations were due to the inhibition of the JAKs phosphorylation as well as the disturbance of the intracellular GSH levels, which in turn led to the glutathionylation of STAT3, thus inhibiting its phosphorylation. Once again, the reduced form of costunolide, lacking the α,β-unsaturated carbonyl, failed to inhibit the activation of STAT3 [40].

In another study comprising both costunolide and its derivative dihydrocostunolide, the α-methylene-γ-lactone proved its importance in the bioactivity of the SL structure once more. Costunolide was proposed for the treatment of psoriasis, as it is able to block the pro-inflammatory effects of IFN-γ and IL-22 in primary human keratinocytes through the inhibition of STAT1 and STAT3 activation. The compound also prevented epidermal hyperproliferation caused by an apoptosis-resistant phenotype [41]. In this case, the pro-oxidant effect of costunolide, caused by conjugation with GSH, was explored as the key aspect for activating the anti-proliferative and pro-apoptotic pathways that are crucial in counteracting the psoriatic phenotype [41].

AP-1 is another transcription factor upon which costunolide exerts its anti-inflammatory effect by inhibiting its DNA-binding activity as well the MAPKs signaling pathway that mediates its activation [42].

In IL-1β-elicited primary rat chondrocytes, costunolide prevented the expression of cytokines, iNOS, COX-2, and matrix metalloproteinases (MMPs), while upregulating collagen II and the transcription factor SRY-box transcrition factor (SOX)-9 (crucial for chondrocyte proliferation and differentiation). Such results were a consequence of the suppression of the NF-κB and Wnt/β-catenin pathways, that are both involved in bone metabolism and reconstruction [43]. These results were complemented by an in vivo attenuation of cartilage degeneration in rat joints, and costunolide was proposed as a promising agent to treat osteoarthritis [43].

An effect of costunolide against inflammatory angiogenesis, which is related to tumor growth and metastasis, was demonstrated in mice [44]. In addition to the attenuation of fibrovascular tissue (hemoglobin content and collagen deposition), along with reduced macrophage and neutrophil recruitment, the compound decreased inflammatory, angiogenic, and fibrogenic mediators, including the vascular endothelial growth factor (VEGF) and the transforming growth factor (TGF)-β [44].

#### 2.3.3. Other Germacranolides

Research interest in SLs is evolving as the biological relevance of this class of compounds is growing, and underexplored germacranolides are emerging as promising novel anti-inflammatory agents. These compounds act in several pathways leading to the activation of different transcription factors that ultimately result in the expression of inflammation-related proteins.

Eupatolide (Figure 4) is a germacranolide that inhibits the activation of both the MAPKs and protein kinase B (Akt) pathways, as well as the phosphorylation of IκBα and p65, thus suppressing the NF-κB and AP-1 transcription factors, which in turn leads to the decreased expression of iNOS and COX-2 along with the resulting NO and PGE_2_ [45]. These outcomes might be a consequence of the eupatolide-induced proteasomal degradation of the tumor necrosis factor receptor (TNFR)-associated factor 6 (TRAF6), which mediates the activation of signaling pathways, including NF-κB and MAPKs [45].

Onopordopicrin (Figure 4), isolated from *Onopordum Illyricum* L., revealed its potential as a potent inhibitor of NF-κB and STAT3 while promoting the activation of Nrf2, an agent of antioxidant defense [46].

In addition to the interference with the transcription factors involved in inflammation, advanced research on different mechanisms of action is also being conducted. Recently, deoxyelephantopin (Figure 4), another germacranolide, has been implicated in a novel strategy to treat inflammatory diseases [47]. This compound decreased macrophage activation and prevented sepsis-mediated death in mice by attenuating aerobic glycolysis at the transcription and protein expression levels of several key glycolytic enzymes [47]. The connection to inflammation lies in the fact that activated immune cells reprogram their energy metabolism from oxidative phosphorylation to aerobic glycolysis, a phenomenon equivalent to the Warburg effect of tumor cells.

Deoxyelephantopin dose-dependently inhibited NO in the LPS-induced murine macrophage cell line RAW 264.7. This decrease was due to the downregulation of the iNOS mRNA and protein levels in macrophages. The compound was also able to decrease the amounts of COX-2 at the mRNA and protein levels while reducing the production of TNF-α and IL-6 back to basal levels [48]. These results were consistent with NF-κB inhibition and the authors confirmed that deoxyelephantopin could prevent the nuclear translocation of the p65 subunit by suppressing the phosphorylation and degradation of IκB, which maintains the nuclear transcription factor inactive in the cytosol, ultimately inhibiting the production of pro-inflammatory cytokines [48]. Deoxyelephantopin was also able to suppress, in vivo, the activation of the IL6/STAT3 pathway and JNK1 and JNK2 in mice liver cells, inhibiting the production of pro-inflammatory cytokines. Moreover, the protein levels of the suppressor of cytokine signaling (SOCS)-3, which is a key component of IL-6 negative regulation, were decreased in deoxyelephantopin treatment of an LPS/D-Galactosamine-induced hepatic inflammation model in mice. Deoxyelephantopin could also attenuate the levels of TNF-α and IL-6 in mice serum, as well as effectively reduce the activity of the iNOS and COX-2 proteins in the hepatic inflammation mice model [48].

### 2.4. Guaianolides

Guaianolides recently isolated from *Ormenis mixta* L. and characterized as 2,3-epoxy-1,4,10-trihydroxyguaian-12,6α-olide diastereoisomers, revealed an anti-inflammatory potential through the inhibition of NO release and COX-2 expression in murine macrophages treated with LPS [49].

**Figure 6:**
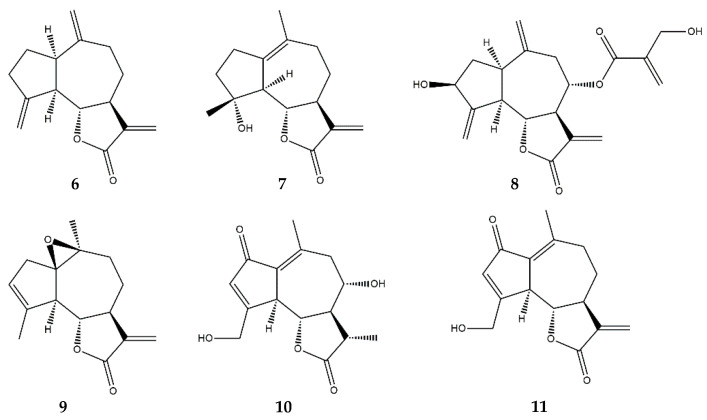
Structures of addressed guaianolides. **6**—Dehydrocostuslactone; **7**—Micheliolide; **8**—Cynaropicrin; **9**—Arglabin; **10**—11β,13-dihydrolactucin; **11**—8-deoxylactucin.

Dehydrocostuslactone (Figure 6) exerts its anti-inflammatory effects through the inhibition of several inflammatory pathways. The treatment of THP-1 cells with dehydrocostuslactone showed its ability to inhibit the activation of the IL-6/STAT3 pathway. Moreover, it suggested that dehydrocostuslactone was able to interact directly with the cellular glutathione content. This interaction created intracellular oxidative stress, leading to the inhibition of STAT3 tyrosine-phosphorylation in IL-6-induced cells, resulting in a downregulation of the expression of genes involved in inflammatory processes, in particular, MCP-1, the C-X-C motif chemokine ligand 10 (CXCL10), and the intracellular adhesion molecule-1 (ICAM-1), with an EC_50_ of 10 µM [40,41]. The ability of dehydrocostuslactone to suppress the tyrosine-phosphorylation of STAT3 suggests that this compound may interfere with the functions of upstream JAK kinases, associated with a portion of the IL-6 receptor. Additionally, dehydrocostuslactone also interfered with IL-22/STAT3 in keratinocytes, resulting in the downregulation of inflammatory genes. The most sensitive genes to the action of this SL were those transcriptionally regulated by STAT3 and whose regulation is driven by the extracellular signal-regulated kinase (ERK) 1 [41].

In a dextran sulfate sodium (DSS)-induced colitis in mice, dehydrocostuslactone (20 mg/kg/day, orally administrated) reduced the quantity of inflammatory cytokines, such as TNF-α, IL-1β, MCP-1, myeloperoxidase (MPO), superoxidase dismutase (SOD), IL-6, IL-17, and IL-23, and once again downregulated the IL-6/STAT3 inflammatory signaling pathway, thus alleviating the colorectal damage caused by DSS [40,41,50]. The decreased activity of this pathway is related to the further downregulation of other inflammatory mediators, such as iNOS and COX-2 [50].

In a different study, dehydrocostuslactone (20 µM) inhibited both the NF-κB pathway and the interferon regulatory factor 3 (IRF3) in LPS-stimulated murine macrophages RAW 264.7 [51], with both transcription factors being regulated upstream by the activation of the Toll-like receptors myeloid differentiation primary response 88 (MyD88) and Toll-interleukin-1 receptor domain-containing adapter- inducing interferon-β (TRIF)-dependent signaling pathways. By suppressing these receptors, dehydrocostuslactone downregulated NF-κB and IRF3, consequently preventing the expression of their target genes including COX-2, INF-β, and the interferon gamma-induced protein-10 (IP-10). Moreover, the treatment of LPS-challenged macrophages with dehydrocostuslactone leads to the suppression of IκBα degradation, strengthening the NF-κB inhibition [51].

Micheliolide (Figure 6), another guaianolide, may pose a therapeutic benefit for the treatment of neurodegenerative disorders via the inhibition of LPS-induced iNOS and COX-2 protein expression in BV2 microglial cells [52]. The compound (10 µM) also demonstrated the ability to attenuate, at the transcriptional level, the expression of multiple pro-inflammatory mediators, namely, TNF-α, IL-6, IL-1β, COX-2, and iNOS, all the genes of which are regulated by the activation of the NF-κB transcription factor and the Akt pathway [52]. The authors verified that micheliolide could block the NF-κB p65 subunit nuclear translocation, maintaining the transcription factor inactive in the cytosol [52,53]. In the same study, micheliolide was shown to exert anti-inflammatory activity by inhibiting the activation of MAPKs, including JNK, p38, and ERK1/2, and phosphatidyl inositol 3-kinase (PI3K)/Akt. Both of these pathways culminate in the activation of NF-κB, which underlines the ability of this compound to inhibit this transcription factor and further downregulate the NF-κB-dependent inflammation players [52].

In a different study, micheliolide proved its ability to decrease inflammatory cytokine production in murine macrophages RAW 264.7, human dendritic cells, and monocytes. The authors demonstrated that micheliolide inhibited the LPS-induced activation of NF-κB and the PI3K/Akt pathway [54].

In vivo studies also demonstrated that pre-treatment with micheliolide, diluted in the drinking water of mice, five days before inflammatory stimuli with DSS, was able to reduce neutrophil and lymphocyte infiltration, attenuating the severity of colitis and the inflammatory damage to the colon tissue. The authors further verified that the administration of micheliolide strongly inhibited IL-6, TNF-α, and IL-1β expression in a murine model of DSS-induced colitis [53].

In an acute peritonitis mouse model, micheliolide (20 mg/kg, intradermal injection) was able to reduce the secretion of IL-6, TNF-α, IL-1β, and MCP-1, resulting in a decreased inflammatory state [54,55]. In a collagen-induced arthritis mouse model, micheliolide, administered intraperitoneally, reduced paw swelling and suppressed the degeneration of articular cartilage, whilst also decreasing the levels of several inflammatory mediators such as the tissue inhibitor of metalloproteinases (TIMP)-1, macrophage colony-stimulating factor (M-CSF), ICAM-1, and INF-γ, thereby reducing the proliferation, adhesion, and infiltration of leukocytes into the affected area [55].

The guaianolide cynaropicrin (Figure 6) possesses anti-inflammatory properties, strongly inhibiting TNF-α release from LPS-stimulated murine RAW 264.7 macrophages, and differentiated human macrophages (U937 cells). Aside from TNF-α inhibition, the compound was also effective in reducing the release of NO from RAW 264.7 macrophages stimulated with LPS and IFN-γ, in a dose-dependent manner [56]. Cynaropicrin also suppressed the proliferation of CD4^+^, CD8^+^ T-, and B- lymphocytes [56].

Arglabin (Figure 6) was described as able to attenuate the overexpression of inflammatory mediators with a decrease in the mRNA levels of NF-κB-regulated genes, such as COX-2, iNOS, and IL-1β in peritoneal mouse macrophages [57]. In vivo, arglabin (2.5 ng/g, intraperitoneal injection, twice daily for thirteen days) inhibited the nucleotide-binding oligomeriztion domain (NOD)-like receptor family pyrin domain-containing 3 (NLRP3) inflammasome activity and significantly reduced the production of the cytokines IL-1α, IL-1β, and IL-18, leading to a reduction in the atherosclerotic lesions in apolipoprotein E (apoE)-deficient mice with an EC_50_ of 10 nM [57].

11β,13-dihydrolactucin (Figure 6) has been revealed as possessing anti-inflammatory potential by reducing the activity of the calcineurin/calcineurin-responsive zinc finger-1 (Crz1) pathway, which is the yeast orthologue of the human calcineurin/NFAT pathway. Calcineurin is highly conserved between eukaryotes making this an optimal model for screening potential anti-inflammatory compounds [58]. 11β,13-dihydrolactucin reduced the activation of the pathway with an IC_50_ of 2.35 µM. Further analysis demonstrated that the compound inhibited the nuclear translocation of Crz1, which remained inactive in the cytosol, in the presence of inflammatory stimuli [58].

8-deoxylactucin (Figure 6) has been described as the most effective SL in a chicory extract. The compound exerts its anti-inflammatory activity by inhibiting COX-2 protein and further downregulating PGE_2_ in human colorectal cancer cells HT29 [59].

### 2.5. Eudesmanolides

**Figure 7:**
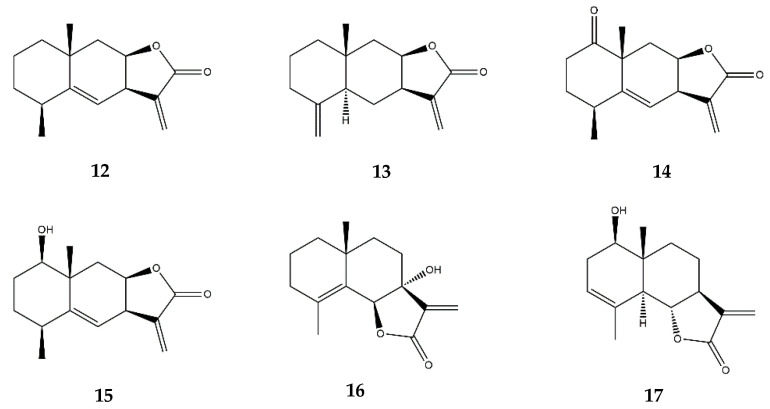
Structures of addressed eudesmanolides. **12**—Alantolactone; **13**—Isoalantolactone; **14**—JEUD-38; **15**—1β-hydroxyalantolactone; **16**—7-hydroxyfrullanolide; **17**—Santamarin.

Alantolactone and isoalantolactone (Figure 7) isolated from *Inula helenium* inhibited the TNF-α-induced activation of the NF-κB and MAPK pathways in mouse endothelial b.End3 cells, suppressed the expression of MMP-3, MCP-1, and IL-1 in synovial fibroblasts, and decreased the expression of IL-1, IL-6, and iNOS in murine macrophages RAW 264.7 [26]. In vivo, alantolactone (orally administered, 50 mg/kg) could also alleviate the arthritic severity and paw swelling in either the developing or the developed phases of adjuvant and collagen-induced arthritis in a mice model [26].

In another study, alantolactone was able to inhibit iNOS and COX-2 at both the mRNA and the protein levels. Furthermore, alantolactone reduced the production of pro-inflammatory markers such as NO, PGE_2_, and TNF-α [60]. Regarding the NF-κB pathway, alantolactone not only inhibited the DNA-binding of the NF-κB p65 subunit, but also suppressed IκB kinase (IKK) phosphorylation, resulting in the blockage of the IκB degradation and the subsequent activation of the NF-κB transcriptional factor [60]. The authors concluded that these effects might occur due to inhibition of the MyD88 and the Toll-interleukin-1 receptor domain-containing adapter protein (TIRAP), an upstream signaling molecule involved in IKK and MAPK activation, in LPS-stimulated macrophages [60].

In an in vitro intestinal inflammation model, alantolactone inhibited NF-κB nuclear translocation and dose-dependently activated the human pregnane X receptor (hPXR), a key regulator gene in IBD pathogenesis [61]. hPXR inhibits NF-κB-driven gene expression. However, NF-κB can also regulate hPXR-driven gene expression. The authors demonstrated that alantolactone directly interacts with the hPXR, thus enhancing the inhibition of the NF-κB pathway, which suggests that the anti-inflammatory effect of alantolactone could be partially driven by the activation of hPXR [61]. In an in vivo model of intestinal inflammation, the oral administration of alantolactone (50 mg/kg) significantly ameliorated the clinical symptoms of DSS-induced colitis in mice by lowering the release of pro-inflammatory mediators such as iNOS, ICAM-1, COX-2, TNF-α, INF-γ, and IL-6 to basal levels [61].

Isoalantolactone (Figure 7), a variant of alantolactone, similarly to its parental compound was also found to inhibit, in vitro, the TNF-α-stimulated activation of the NF-κB and MAPKs pathways in b.End3 cells, suppress the expression of MMP-3, MCP-1, and IL-1 in TNF-α-stimulated synovial fibroblasts, and reduce IL-1 and IL-6 production in LPS-stimulated murine RAW 264.7 macrophages [26].

1β-hydroxyalantolactone (Figure 7) peculiarly demonstrates its anti-inflammatory effects by inhibiting the ubiquitin-conjugating enzyme H5 (UbcH5) during TNF-α-induced NF-κB activation. This enzyme mediates the ubiquitination of several important signaling proteins upstream of IKK, resulting in a non-phosphorylated IKK that is in turn unable to degrade IκB, thereby preventing the further activation of NF-κB and ultimately suppressing the downstream pro-inflammatory gene expression [62].

JEUD-38 (Figure 7) is a recently discovered SL from *Inula japonica* L., a plant from the Asteraceae family, which inhibited the nuclear translocation of p65 by impeding IκBα phosphorylation and degradation in murine RAW264.7 macrophages [63]. In addition, JEUD-38 inhibited the LPS-stimulated phosphorylation of MAPKs, including ERK1, ERK2, JNK, and p38 kinases, further strengthening its inhibitory effects on NF-κB suppression [63].

7-hydroxyfrullanolide (Figure 7) suppressed the LPS-induced NF-κB-related transcripts in human PMBCs and in freshly collected synovial cells from rheumatoid arthritis patients, by inhibiting the nuclear translocation of NF-κB through the inhibition of IKK phosphorylation in THP-1 cells [64]. Since NF-κB regulates the transcription of adhesion molecules, the authors further explored the expression of ICAM-1, vascular cell adhesion molecule (VCAM)-1, and E-selectin in LPS-stimulated endothelial cells, concluding that treatment with 7-hydroxyfrullanolide suppressed their production and also inhibited monocyte adhesion [64]. 7-hydroxyfrullanolide was also tested in different animal inflammation models, in which oral administration dose-dependently diminished the induced and spontaneous production of TNF-α along with IL-6 in a BALB/c ear edema model and in DSS-induced colitis in mice [65]. Administration of 7-hydroxyfrullanolide also attenuated rectal bleeding and colonic edema, and diminished the shortening of the colon, as was verified by histological images [65].

Santamarin (Figure 7) inhibited the activation of the iNOS and COX-2 proteins, consequently reducing their respective products, namely NO and PGE_2_ in LPS-stimulated RAW 264.7 cells and murine peritoneal macrophages [66]. Santamarin also reduced TNF-α and IL-1β production by suppressing the phosphorylation and degradation of IκBα, and by blocking the nuclear translocation of p65 in response to LPS in RAW 264.7 macrophages [66]. Additionally, both the mRNA and protein levels of the anti-inflammatory protein HO-1 were upregulated in the presence of Santamarin [66]. HO-1 is primarily regulated at the transcriptional level, and it may be induced by various agents, including the Nfr2 transcription factor. The effects of Santamarin on NO, PGE_2_, and TNF-α production were partially reversed by the usage of an HO-1 inhibitor, suggesting that part of the anti-inflammatory effect demonstrated by Santamarin is related to the induction of HO-1 [66].

### 2.6. Heliangolides

**Figure 8:**
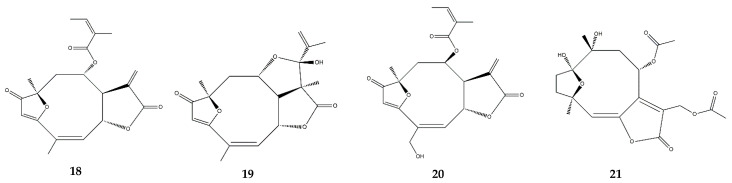
Structures of addressed heliangolides. **18**—Lychnopholide; **19**—Eremantholide C; **20**—Budlein A; **21**—Diacethylpiptocarphol.

In vitro assays demonstrated that lychnopholide (Figure 8) inhibited NO production in J774A.1 macrophages stimulated by INF-γ and LPS, and increased the production of IL-10, an anti-inflammatory cytokine [67]. In vivo, lychnopholide reduced a carrageenan-induced paw edema when administered topically in ointment formulation at a concentration of 1% [67].

Eremantholide C (Figure 8) was able to reduce TNF-α release while increasing IL-10 production in J774A.1 macrophages [67]. In vivo, eremantholide reduced the carrageenan-induced paw edema, possibly due to TNF-α inhibition and the induction of IL-10 production when applied topically in ointment formulation at a concentration of 1% [67].

Budlein A (Figure 8), a furanoheliangolide, possesses anti-inflammatory activities related to the inhibition of pro-inflammatory cytokines and neutrophil recruitment. In vitro, budlein A reduced the NF-κB activity in murine macrophages RAW 264.7 [68]. Treatment with budlein A decreased neutrophil recruitment in models of innate immune response and inhibited the LPS-induced expression of adhesion molecules such as E-selectin, ICAM-1, and VCAM-1, all of which are correlated with NF-κB inhibition [68]. The authors also showed that budlein A reduces lymphocyte proliferation and the release of IL-2 and INF-γ. The effect of budlein A was also evaluated in antigen-induced arthritis in mice, in which it dose-dependently inhibited IL-33, TNF-α, IL-1β, and COX-2 mRNA expression [68]. In another study, budlein A inhibited carrageenan-induced neutrophil migration to the peritoneal cavity, neutrophil migration to the paw skin tissue, paw edema, and mechanical hypernociception [69]. Moreover, the treatment inhibited the mechanical hypernociception induced by TNF-α and IL-1β but not the hypernociception caused by PGE_2_ or dopamine [69]. In a similar study, budlein A was tested as a therapeutic agent against gout arthritis in a murine model, in which it prevented NF-κB activation by inhibiting TNF-α production, and attenuated neutrophil recruitment [70]. An in vitro analysis demonstrated that, in addition to TNF-α inhibition, budlein A reduced the production/maturation of IL-1β, suggesting that it may not only inhibit the NF-κB pathway but also interfere with the assembly of inflammasome NLRP3 in macrophages [70].

The intraperitoneal injection of diacethylpiptocarphol (Figure 8), a heliangolide isolated from *Vernonia scorpioides* L., in a DSS-induced colitis mouse model significantly decreased immune cell infiltration, tissue damage, and TNF-α release, while enhancing the production of TGF-β, which is involved in tissue remodeling. This newly isolated compound displayed results identical to those of parthenolide and the anti-inflammatory drug dexamethasone [71].

### 2.7. Pseudoguaianolides

**Figure 9:**
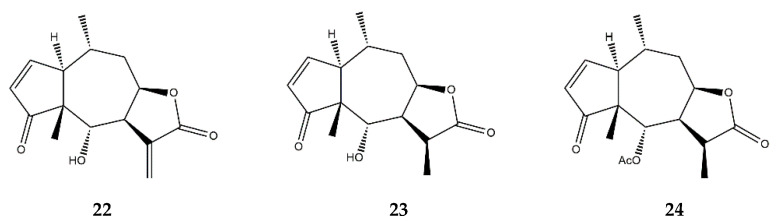
Structures of the addressed pseudoguaianolides: **22**—helenalin; **23**—11α,13-dihydrohelenalin; **24**—11α,13-dihydrohelenalin acetate.

Helenalin (Figure 9) is a known potent anti-inflammatory compound that can inhibit NF-κB activation in T- and B-lymphocytes, as well as in epithelial cells [72]. Whilst helenalin cannot prevent IκBα degradation nor NF-κB nuclear translocation, the compound can downregulate the pro-inflammatory NF-κB-driven gene expression by preventing the DNA-binding of the NF-κB p65 subunit since it modifies the active p65 by reacting with its free cysteines through Michael-type addition, thereby irreversibly alkylating its structure [72,73].

Helenalin also demonstrates immunosuppressive effects in THP-1 cells, by decreasing cytokine release, namely the granulocyte-macrophage colony-stimulating factor (GM-CSF), IL-1α, IL-19, IL-23, and MCP-3. Since IL-19 plays a role in promoting the release of IL-6 and TNF-α, its suppression by helenalin is an important anti-inflammatory strategy [74,75]. The capacity of helenalin to inhibit the DNA-binding of the p65 subunit has been correlated with an increase in cellular death triggered by the mitochondrial pathway of apoptosis in CD4+ T-cells, which is a relevant anti-inflammatory effect [74,76]. T-cells that survive the exposure to helenalin undergo proliferation inhibition by the induction of G2/M cell cycle arrest [74]. Helenalin was also able to suppress the nuclear translocation of NFAT in activated CD4^+^ T-cells, by decreasing the production of IL-2 in such lymphocytes [23,74].

11α,13-dihydrohelenalin (Figure 9) demonstrated the ability to inhibit DNA-binding as well as NF-κB activation, and although its variant 11α,13-dihydrohelenalin acetate was unable to prevent the DNA-binding of NF-κB, it significantly affected MAPKs, a result not verified for the parent compound [77]. Besides the NF-κB pathway and immune cell proliferation inhibition, helenalin and 11α,13-dihydrohelenalin acetate also display anti-inflammatory effects in the arachidonic acid pathway [78]. Helenalin and, to a lesser extent, 11α,13-dihydrohelenalin acetate, demonstrated the ability to inhibit both leukotriene C_4_ synthase and 5-LOX in a concentration-dependent manner [78].

### 2.8. Other SL Subclasses

Vlasouliolides isolated from *Vladimiria souliei* L., consisting of rare SL dimeric structures with 32 carbons, presented an anti-inflammatory effect by inhibiting the phosphorylation of the NF-κB subunit p65 and preventing NO production in LPS-elicited murine macrophages [79].

**Figure 10:**
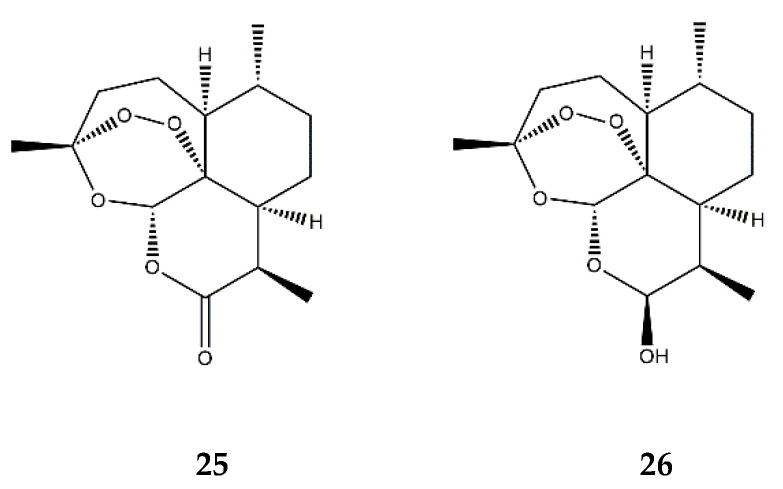
Structures of **25**—artemisinin and **26**—dihydroartemisinin.

It has also been described that artemisinin (Figure 10), a renowned SL due to its action against *Plasmodium falciparum* and therefore used in the treatment of malaria, significantly decreases the adhesion of monocytes to TNF-α-stimulated human umbilical vein endothelial cells (HUVECs), in a dose-dependent manner. The compound was also found to suppress the mRNA and protein levels of ICAM-1 and VCAM-1, leading to an attenuation of monocyte adhesion to HUVECs, which could be explained by the inhibition of the NF-κB signaling pathway [80]. Indeed, treatment with artemisinin also significantly increased the cytosolic levels of IκBα, the protein that maintains NF-κB in its inactive state, thus downregulating the expression of pro-inflammatory genes in the TNF-α-stimulated HUVECs [80]. Moreover, this SL was also found to inhibit the nuclear translocation of the activated p65 subunit of NF-κB and inhibit the ERK1 and ERK2 members of the MAPKs [80].

Dihydroartemisinin (Figure 10) is a semi-synthetic derivate of artemisinin and has been described as having anti-inflammatory effects in murine RAW 264.7 macrophages with a dose-dependent decrease in the PMA-induced COX-2 expression and subsequent PGE_2_ production [81]. These observations could be a result of dihydroartemisinin’s inhibiting effects on the NF-κB, AP-1, and C/EBP pathways. Similar to the parent compound artemisinin, dihydroartemisinin also affected the MAPKs, inhibiting the activity of JNK, ERK1, ERK2, and p38 kinases [81].

A study conducted with 17 natural SLs belonging to the germacranolide, guaianolide, pseudoguaianolide, and eudesmanolide subclasses revealed that the bioactivities of these compounds are not always mediated by α,β-unsaturated carbonyl alkylation. In fact, the inhibition of isolated human neutrophil elastase, a protease implicated in the pathogenesis of several inflammatory diseases, was achieved by non-covalent interaction with the catalytic site, requiring the specific structural features of SLs such as a carbonyl group surrounded by hydroxy groups at a certain distance. Nonetheless, human neutrophil elastase was not considered a direct target for SLs, since most compounds require a high concentration to accomplish the inhibition of the enzyme [82].

Another set of varied SLs, including germacranolides, pseudoguaianolides, eudesmanolides, and heliangolides, was shown to effectively inhibit the activation of T-cells in whole blood samples, a probable consequence of the decreased expression of IL-2, a mediator of lymphocyte proliferation [35]. Interestingly, SLs presenting with only one α,β-unsaturated carbonyl moiety (monofunctional) were more effective than those with two of these functional groups (bifunctional) [35]. A reasonable explanation for this is the fact that the latter are more reactive and tend to bind to unspecific targets, such as albumin, thereby rendering them unavailable to react with the desired target. Less reactive SLs preferentially bind with targets for which they have the highest affinity rather than being trapped with matrix proteins.

The described anti-inflammatory outcomes of isolated SLs in the abovementioned models are summarized in Table 2.

## 3. Structure–Activity Relationship of SLs

Structure–activity relationship (SAR) studies have uncovered valuable knowledge on how the specific structural elements of SLs can influence their bioactivity [10,12,83]. The α,β-unsaturated carbonyl structures (α-methylene-γ-lactone or α,β-unsaturated cyclopentenone) are the most relevant structural features for the bioactivity of SLs, with the other functional groups essentially modifying their potency by steric and chemical influences (lipophilicity, molecular geometry, conformational flexibility, and the chemical environment surrounding the functional groups of the SL and the biological target) [1,84]. However, SLs containing more than one reactive center may have the capability of interacting with a broader variety of biological targets. Indeed, SLs combining an α-methylene-γ-lactone with an epoxide can react with the sulfhydryl groups through the α-methylene-γ-lactone moiety, as well as with the hydroxyl and amine groups through the epoxide ring [10]. Hence, the combination of potentially reactive sites with different reactivities provides versatility in terms of biological targets, an angle that may be exploited to maximize the desired bioactivity of SLs [10]. The synthesis of semi-synthetic analogs helps researchers understand how specific modifications to the parent compound can affect bioactivity and cytotoxicity, two criteria that need to be carefully balanced. For instance, the synthesis of eudesmanolide SLs from costunolide rendered two promising compounds in terms of TNF-α inhibition; however, the presence of an epoxide ring in one of the structures led to lower cytotoxicity [85].

Wang et al. [86] reviewed the structure–activity relationship of several α-santonin synthetic derivatives and concluded that the conserved α-methylene-γ-lactone moiety was of great importance for the immunomodulatory activity and cytotoxicity. Conversely, the modification of the lactone ring with amino-derivatives led to a more moderate cytotoxic activity, whereas the presence of an endoperoxide unit in the structure increased toxicity [86]. The introduction of aromatic thiol derivatives substituted with electronegative atoms (halogens) in the lactone ring increased the binding affinity with the p65 subunit of NF-κB, due to the formation of an additional hydrogen-bond with key amino acids [86]. In addition, the introduction of benzoic or cinnamic acid side chains can favor the inactivation of UbcH5c, a key ubiquitin-conjugating enzyme that plays a role in the activation of NF-κB, and the prevention of NO release [86]. Moreover, the introduction of O-benzyl ethers in α-santonin derivatives improved docking with COX-2, due to the interaction of the phenyl group with a selectivity pocket in the catalytic site [87]. However, if the substitute is too bulky, the affinity with the enzyme decreases [87]. Another molecular docking study conducted for a guaianolide isolated from *Cyathocline purpurea* L. revealed the selectivity of the compound for COX-2 over COX-1 [88].

In a study involving 1β-hydroxyalantolactone and ivangustin, the introduction of aliphatic or aromatic groups while maintaining the α-methylene-γ-lactone resulted in a decreased cytotoxicity [89]. In the same work, a molecular modeling approach revealed that the oxidation of hydroxyl into a carbonyl function in the structure of 1β-hydroxyalantolactone improved docking with p65 [89]. Accordingly, molecular docking of ambrosin with p65 revealed that two H-bonds can form due to the presence of two carbonyl functions in the structure [90]. From what was demonstrated in the aforementioned studies, the interactions between the SLs and the target proteins are stabilized by alkyl bonds through the α-methylene-γ-lactone, as well as van der Waals forces through other functional groups present in the structure. In fact, the cytotoxicity of the SLs greatly depends on the number of alkylating centers; nonetheless, molecular conformation and noncovalent interactions before alkylation may also be determinant variables [91].

Another useful approach would be to run in silico studies to assess whether a novel compound has similar behavior to that of known drugs or validated molecules. This was the strategy followed by Talhouk et al. [92] that evaluated in silico molecular docking of a novel SL from *Cota palaestina* L. with several known protein targets of parthenolide, including p65, MAPKs, IKK, and STATs. The results allowed the authors to conclude that the studied SL could bind to several protein targets with a calculated binding affinity comparable to that of parthenolide [92].

Enhanced selectivity could also be achieved by introducing structural changes to the scaffold in a way that would direct the compound to the site of action or allow its activation/release through metabolic reactions that specifically take place in the target tissue (e.g., prodrug) [10].

A fact that should also be considered is that different reaction groups may have different reaction kinetics, as was demonstrated in helenanolide-type SLs [93]: helenanolides containing α,β-unsaturated cyclopentenones reacted with GSH in a rapid but reversible manner, whereas those containing α-methylene-γ-lactones reacted at a slower rate but in an irreversible way, leading to permanent deactivation. This suggests that although SLs containing α-methylene-γ-lactones may eventually suffer irreversible deactivation after reacting with a nucleophile, these compounds have slower reaction kinetics and thus might have more time to react with biological targets than SLs with α,β-unsaturated cyclopentenones [93]. Conversely, SLs displaying α,β-unsaturated cyclopentenones may react rapidly, but these modifications can be reversed and the SLs may once again become available to interact with other targets [93].

This knowledge of the structural features of SLs, and the respective consequences for bioactivity, could aid the optimization of novel lead compounds by guiding the synthesis of molecules with the desired bioactivity. For instance, parthenolide [94,95] and arglabin [96] derivatives have already been developed, presenting improved bioavailability and pharmacological properties while maintaining bioactivity due to a pro-drug mode of action.

An important SAR study comprising of a set of 103 SLs belonging to different structural subclasses was reported by Siedle et al. [91], shedding some light on which molecular descriptors best described the ability of the compounds to prevent NF-κB DNA-binding activity. When considering the whole set of SLs, the number of α,β-unsaturated carbonyl functions and, in particular, the presence of an α-methylene-γ-lactone, were the most relevant variables [91]. Nonetheless, when smaller sets of structurally related SLs were considered, the differential importance of certain molecular features was underlined. Indeed, for SLs with a more flexible structure, such as germacranolides, bioactivity was mostly determined by the number of reactive groups; on the other hand, for more rigid structures such as eudesmanolides, topological parameters were the most relevant [91]. For guaianolides, which possess relatively rigid structures, the polarizability of the molecule was an important descriptor [91]. In sum, an analysis of such a structurally diverse set of compounds could not provide a reasonable quantitative prediction of bioactivity; therefore, it is hard to select an SL subclass as the most promising one for anti-inflammatory purposes, since the molecular descriptors that best describe bioactivity vary between different classes [91].

## 4. Pharmacokinetic Concerns

Although there is significant scientific evidence that SLs have health-promoting effects, there are still some questions concerning the bioavailability of these compounds. The pharmacokinetic profile of a given molecule must always be considered because bioactivity towards a certain target in silico or in vitro does not necessarily translate into drug efficacy in vivo. When a compound is being considered for physiological purposes, one must always ponder whether its molecular structure can reach the desired target in an active form. For instance, in the case of oral administration, the molecule should be resistant to different pH conditions and to the action of several enzymes, such as esterases, that are distributed along the gastrointestinal tract of mammals [97]. Distribution and metabolism issues should also be considered.

Although SLs are generally looked upon as promising drug candidates, due to their bioactivity and drug-likeness (according to Lipinski’s rule of five), some SLs have limited permeability and solubility, as well as a high affinity towards plasma proteins, which may result in a poor bioavailability [98,99]. For instance, it has been hypothesized that monofunctional SLs (with a unique alkylating center) display a better permeation profile and a lower affinity to plasma proteins than multifunctional SLs, which in turn improves bioavailability and efficacy at the desired site of action [8,98].

In a study involving healthy volunteers, Weng et al. [28] evaluated the absorption, metabolism, and excretion of SLs after the ingestion of a Brussels/witloof chicory juice. Although the largest percentage of consumed SLs (mainly lactucin and some derivatives) was recovered in the fecal samples (43.76%), 7.03% was detected in the blood samples and 1.13% in the urine samples, suggesting the systemic distribution of the compounds [28]. The authors also demonstrated that ingested SLs undergo phase II metabolism (glucuronidation and sulfation) and are metabolized by the gut microbiota in healthy humans [28]. These results are consistent with those reported by García et al. [100] for lactucin and lactucopicrin from escarole salad, which underwent dehydroxylation and hydrogenation by gut microbiota, as well as phase II metabolic reactions.

In another work, alantolactone and isoalantolactone were distributed to several organs after the oral administration of a *Radix inulae* L. extract to rats [101].

In silico predictions considering specific structural parameters might also help anticipate whether a compound is likely to cross biological barriers, allowing the selection of the most promising molecules to proceed to more complex in vitro and in vivo experiments. For instance, an in silico study performed with ten SLs predicted which molecules would be more permeable across the intestinal barrier and led to the selection of only six that proceeded to the permeability assessment in an intestinal cell model [58]. In another research study, in silico methods showed that ambrosin had favorable pharmacokinetic properties, such as molecular polar surface area and logP, to cross the BBB [90].

## 5. Concluding Remarks

**Figure 11:**
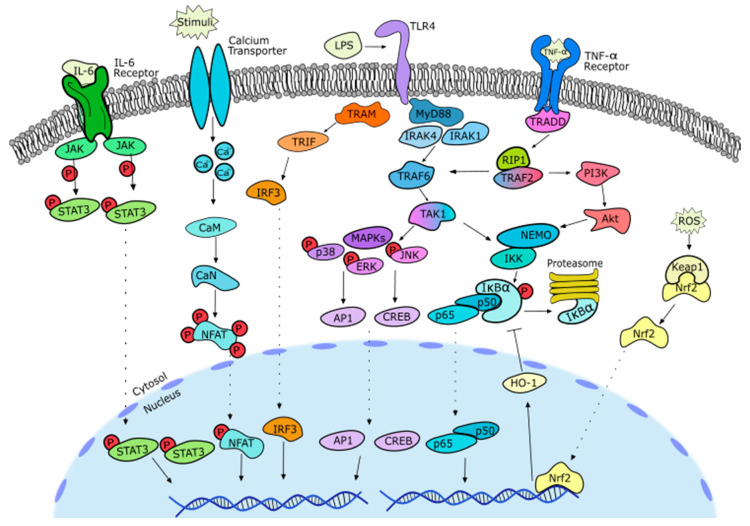
Inflammatory pathways upon which SLs have been described to exert an effect upon. IL-6—interleukin-6; JAK—Janus kinase; P—phosphate group; STAT3—signal transducer and activator of transcription 3; Ca^2+^—calcium ion; CaM—calmodulin; CaN—calcineurin; NFAT—Nuclear factor of activated T-cells; LPS—lipopolysaccharide; TLR4—Toll-like receptor 4; TRIF—Toll-interleukin-1 receptor domain-containing adapter inducing interferon-β; TRAM—TRIF-related adapter molecule; IRF3—interferon regulatory factor; MyD88—myeloid differentiation primary response 88; IRAK1/4—interleukin 1 receptor associated kinase 1/4; TRAF2/6—tumor necrosis factor receptor (TNFR)-associated factor 2/6; TAK1—transforming growth factor-β activated kinase 1; MAPKs—mitogen-activated protein kinases; p38—MAPKs member; Erk—extracellular signal-regulated kinase; JNK—c-Jun N-terminal kinase; AP1—activator protein 1; CREB—cyclic adenosine monophosphate (cAMP) response element-binding protein; TNF-α—tumor necrosis factor-alpha; TRADD—TNF-α-associated death domain protein; RIP1—receptor-interacting protein 1; PI3K—phosphatidylinositol-3-kinase; Akt—protein kinase B; NEMO—Nuclear factor-kappa B essential modulator; IκBα—NF-κB inhibitor alpha kinase; IKK—IκBα kinase; p50/p65—NF-κB protein subunits; HO-1—heme oxygenase-1; ROS—reactive oxygen species; Keap1—Kelch-like ECH-associated protein 1; Nrf2—nuclear factor erythroid 2-related factor 2.

In sum, SLs are promising bioactive compounds that are capable of interacting with several biological targets. These compounds are of particular interest in the field of inflammation since they can inhibit numerous transcription factors responsible for downstream effects such as the release of several pro-inflammatory mediators, including cytokines (IL-1β, TNF-α, IL-6, IL-8, etc.) and enzymes (iNOS, COX-2, kinases, proteases, etc.) (Figure 11). Besides these effects at the level of gene expression, SLs can also directly affect the release or activity of these mediators.

The bioactive potential behind SLs has only recently begun to be uncovered, and the scientific community still has much work to do for SLs to have a recognized clinical efficacy. In particular, a few issues need to be considered; namely, the possibility for toxic or allergic reactions, since many SLs are known to be allergens. The administered dosages must be proven to be safe. Pharmacokinetic profiles must be considered as it is important to assess whether the SLs can cross biological barriers (intestinal, endothelial, BBB, etc.) to reach the intended site of action in physiologically relevant amounts and without significant side effects. Moreover, the possibility that some compounds may not be bioavailable in their original forms due to interactions with plasma proteins, gut microbiota metabolism, or even intracellular glutathione binding, should be considered. The formation of new metabolites that have a higher/lower or a different activity from the respective parental compound must also be contemplated. Possible new approaches for a controlled delivery may need to be developed, implying the need for more studies in more complex in vivo models.

The laboratory development of semi-synthetic SLs should aim at maximizing their anti-inflammatory potential while minimizing cytotoxic side effects. So far, researchers have developed promising semi-synthetic compounds based on the current understanding of the structure–activity relationship. These studies demonstrated that the presence of an α-methylene-γ-lactone moiety is greatly responsible for the anti-inflammatory activity exhibited by SLs. Combining different functional groups could enhance the versatility in terms of biological targets, thus reducing cytotoxicity and exploiting the desired bioactivity of newly synthesized SLs.

The bioactivity within each SL subclass is a consequence of the structural and chemical nature of the molecules. Germacranolides are more flexible than other subclasses and easily adapt the conformation of their functional groups, making them more available to interact with different biological targets. On the other hand, more rigid structures, such as eudesmanolides, are less adaptable to the active sites of targeted proteins, and their shape and topology limit their bioactivity. Considering the vast array of molecular structures and the different targets presented within this review, it is difficult to quantitatively predict which is the most promising SL subclass in terms of anti-inflammatory potential. However, there is already a significant amount of information in the literature to guide the optimization of lead structures, and a promising future for SLs as anti-inflammatory compounds can be envisioned.

Overall, SLs have been used in traditional medicine for centuries and can be regarded as very interesting compounds with a myriad of bioactivities that can be pharmacologically exploited either as drugs or as structural leads for anti-inflammatory drug development. Since SLs are natural compounds frequently found in natural extract preparations, their potential development into functional foods or food supplements should not be dismissed.

## Figures and Tables

**Table 1:** Anti-inflammatory effects of SL-containing natural extracts in different in vitro and in vivo experimental models. The affected inflammatory pathways are described, as well as the experimental outcomes observed in each study, and the extract concentration range tested. ↓—decrease; ↑—increase.

Source	Main SLs	Model		Extract Concentration Range	Inflammatory Pathways	Consequences	References
*Cichorium intybus* L.	Dihydrolactucin, lactucin, deoxylactucin, jacquinelin and dihydrolactucopicrin	In vitro	RAW 264.7 murine macrophages + LPS	IC_50_ (μg/mL): 117 for COX-2; 39 for iNOS; 48 for TNF-α; 22 for IL-1β; 21 for NO	-	↓ COX-2, iNOS, TNF-α, IL-1β, NO	[17]
		In vivo	Paw edema model: Wistar rats + carrageenan (subcutaneous)	50–100 mg/kg (oral administration)	-	↓ paw volume (edema)	
			Arthritis model: Wistar rats + collagen (intravenous)	200 mg/kg (oral administration)	-		
*Artemisia leucodes* L.	Leukomisin and austricin	In vitro	RAW264.7 murine macrophages + LPS	2–100 μg/mL	-	↓ COX-2, iNOS, IL-1β, NO	[18]
			COX-1 and -2 enzymatic assay	45–225 μg/mL		↓ COX-2	
		In vivo	Paw edema model: Wistar rats + carrageenan (subcutaneous)	50–200 mg/kg (oral administration)		↓ paw edema	
			Chronic inflammation model: Wistar rats + cotton implant granuloma test	50 mg/kg (oral administration)		↓ granuloma and inflammatory cell infiltrate	
*Artemisia khorassanica* L.	Unspecified	In vitro	J774A.1 murine macrophages + LPS	10–100 μg/mL	↓ NF-κB	↓ COX-2, PGE_2_, iNOS, NO, TNF-α and IL-1β	[19]
*Artemisia* sps (*A. kopetdaghensis, A. santolina, A. Sieberi, A. Fragrans, A. Absinthium, A. ciniformis*)	Saturated, unsaturated and unusual SLs	In vitro	J774A.1 murine macrophages + LPS	10–100 μg/mL	-	↓ COX-2, PGE_2_, iNOS and NO	[20]
*Eupatorium perfoliatum* L.	Diguaiaperfolin (dimeric guaianolide) and Eupafolin (flavonoid)	In vitro	RAW264.7 murine macrophages + LPS	1–100 μg/mL	-	↓ NO, CSF-3, IL-6, IL-1α, IL-1β, TNF, Chemokine (C-C motif) ligand (CCL)-2, CCL22 and CXCL10	[21]
*Xanthium spinosum* L.	Ziniolide	In vitro	Rat polymorphonuclear leukocytes (PMNLs) + ionophore A23187 and Ca^2+^	0–100 μg/mL	↓ NF-κB and arachidonic acid	↓ 5-LOX	[22]
			Human platelets + ionophore A23187	25–200 μg/mL		↓ COX-1 and 12-LOX; ↑ 15(S)-HETE	
			HeLa cells + Phorbol 12-myristate 13-acetate (PMA)	12.5–100 μg/mL		↓ NF-κB activation	
*Arnica montana* L.	Helenalin and dihydrohelenalin ester derivatives	In vitro	Jurkat T cells + TNF-α or PMA	0.5–10 μL/mL	↓ NF-κB and NFAT	↓ NF-κB and NFAT DNA-binding	[23]
			Human PBMCs from healthy donors + LPS	0.001–10 μL/mL		↓ TNF-α and IL-1β	
*Centaurea* L. species (*C. aphrodisea, C. athoa, C. hyalolepis, C. iberica, C. polyclada*)	SL fraction (athoin, 14-O-acetylathoin and methyl-14-O-acetylathoin-12-oate in C. *athoa*)	In vitro	SW1353 human chondrosarcoma cells + PMA	0–100 μg/mL	↓ NF-κB	↓ NF-κB activity	[24,25]
			RAW264.7 murine macrophages + LPS			↓ NO	
		In vivo	Paw edema model: Wistar rats + carrageenan (subcutaneous)	6.75–50 mg/kg (oral administration)		↓ edema	
*Inula helenium* L	Alantolactone and isoalantolactone	In vitro	bEnd.3 mouse endothelial cells + TNF-α	0.6–2.4 μg/mL	↓ NF-κB and MAPKs	↓NF-κB inhibitor (IκB)-α, NF-κB p65, p38 and c-Jun N-terminal kinase (JNK) phosphorylation	[26]
			RAW264.7 murine macrophages + LPS			↓ IL-1, IL-6 and iNOS	
			Primary synovial fibroblasts from rheumatoid arthritis patients + TNF-α			↓ IL-1, MCP-1 and MMP-3	
		In vivo	Adjuvant-induced mice arthritis model	12.5–50 mg/kg (oral administration)		↓ paw swelling	
			Collagen-induced mice arthritis model				
*Arctium lappa* L.	Onopordopicrin	In vivo	Colitis model: Wistar rats + Trinitrobenzene Sulfonic Acid (TNBS) (enteral instillation)	25–50 mg/kg (oral administration)	-	↓ TNF-α and COX-2; ↓ histological damage; ↓ mucin layer loss; ↓ neutrophil infiltration	[27]
*Vernonia scorpioides* L.	Diacethylpiptocarphol and related hirsutinolides	In vivo	Acute ear edema model: Swiss mice + 12-O-tetradecanoylphorbol acetate (TPA) (topical)	0.003–1 mg (topical)	↓ NF-κB	↓ neutrophil infiltration, edema and epidermal proliferation	[29]
			Chronic ear edema model: Swiss mice + arachidonic acid (topical) or croton oil (topical)	1 mg (topical)			

**Table 2:** Anti-inflammatory effects of SLs belonging to the different SL subclasses in varied in vitro and in vivo experimental models. The affected inflammatory pathways are described, as well as the experimental outcomes observed in each study. ↓—decrease; ↑—increase.

SL Subclass	Compound Name (ID Number)	Model		Compound Concentration Ranges Tested	Inflammatory Pathways	Consequences	References
Germacranolides	Parthenolide (1)	In vitro	Rat aortic smooth muscle cells + LPS/IFN-γ	3–30 μM	↓ NF-κB	↓ iNOS and NO release	[31]
		In vitro	BV2 mouse microglia + LPS	5 μM	↓ NF-κB	↓ IL-6 and TNF-α	[32]
		In vitro	Rat primary neural-glial cells + LPS	403 μM	↓ NF-κB, NF-IL6, Nrf-1 and PGC1α	↓ IL-6 and TNF-α	[33]
		In vivo	Wistar rats + LPS (intraperitoneal injection)	1 mg/kg (intraperitoneal injection)		↓ IL-6 and TNF-α in plasma; ↓ COX-2, NF-IL6, SOCS3, IκBα and Tribbles pseudokinase 1 (Trib1) in hypothalamus; ↓ fever	
		In vitro	Jurkat T cells and primary peripheral human T cells + PMA/ionomycin or anti-CD3/CD28	1.25–5 μM	↓ NF-κB and AP-1	↓ IL-4, IL-2 and IFN-γ	[34]
			Primary peripheral human T cells + PMA/ionomycin or anti-CD3/CD28				
		In vitro	Blood from healthy donors + PMA/ionomycin	10–500 μM	-	↓ IL-2; ↓ T-lymphocyte activation	[35]
		In vitro	Human THP-1 monocytes + LPS	0.75–12 μM	↓ NF-κB and MAPKs	↓ IL-6, TNF-α, IL-1β, IL-8, IL-18 and NO; ↓ iNOS, TLR4 and TRAF6	[36]
			Human primary monocytes + LPS				
		In vivo	Hindpaw edema model: Holtzman rats + carrageenan (subcutaneous injection)	5–20 mg/kg (intraperitoneal injection)	-	↓ Hyperalgesia and edema	[37]
		In vitro	HepG2 human hepatocytes + IL-6, oncostatin M or leukemia inhibitory factor	5 μM	↓ STAT3 and JAKs	↓ STAT3 phosphorylation, dimerization and activity	[39]
	Costunolide (2)	In vitro	Human THP-1 monocytes + IL-6	6–25 μM	↓ IL-6/STAT3 and JAKs	↓ MCP-1, CXCL10, ICAM-1; ↓ STAT3 phosphorylation and DNA-binding activity; ↓ Intracellular GSH	[40]
		In vitro	RAW264.7 murine macrophages + LPS	0.1–1 μM	↑ Nrf-2; ↓ NF-κB	↑ HO-1; ↓ IL-6 and TNF-α	[38]
		In vitro	Human keratinocytes from healthy donors + IL-22, IFN-γ or TNF-α	12.5 μM	↓ STAT3 and STAT1	↓ Intracellular GSH; ↓ CCL2, CXCL10, ICAM-1 and SOCS3; ↑ Epidermal growth factor receptor (EGFR) and Erk1/2	[41]
		In vitro	RAW264.7 murine macrophages + LPS	0.1–3 μM	↓ AP-1 and MAPKs	↓ IL-1β	[42]
		In vitro	Primary rat chondrocytes + IL-1β	2–6 μM	↓ NF-κB and Wnt/β-catenin; ↑ SOX-9	↓ MMP-3, MMP-9, MMP-13, iNOS, COX-2 and IL-6; ↑ collagen II	[43]
		In vivo	Sprague-Dawley rats (surgically induced osteoarthritis model)	6 μM (intra-articular injection)		attenuation of cartilage degeneration	
		In vivo	Angiogenesis model: Swiss albino mice + polyester-polyurethane sponge implants	5–20 mg/kg (cannula)	-	↓ Angiogenesis, macrophage and neutrophil accumulation, and collagen deposition; ↓ IL-1β, IL-6, IL-17, TNF-α, TGF-β; ↑ IL-10	[44]
	Eupatolide (3)	In vitro	RAW264.7 murine macrophages + LPS	0.1–10 μM	↓ NF-κB, AP-1, MAPKs, Akt	↓ COX-2, PGE_2_, iNOS, NO and TRAF6	[45]
			Human embryonic kidney (HEK)-293 cells + LPS			↑ proteossomal degradation of TRAF6	
	Onopordopicrin (4)	In vitro	NIH-3T3 cell line + TNF-α	IC_50_ (μM): 8.6 for NF-κB; 15.3 for STAT3; EC_50_ (μM): 2.2 for Nrf-2	↓ NF-κB and STAT3; ↑ Nrf-2	↓ NF-κB activity	[46]
			HeLa cell line + IFN-γ			↓ STAT3 activity	
			HaCaT keratinocytes			↑ Nrf-2 activity	
	Deoxyelephantopin (5)	In vitro	RAW264.7 murine macrophages + LPS	2.5–10 μM	-	↓ high mobility group box (HMGB) 1, pyruvate kinase M2 (PKM2), glucose transporter 1 (GLUT1), lactate dehydrogenase A (LDHA) and phosphoinositide-dependent kinase 1 (PDK1) and IL-1β	[47]
		In vivo	C57BL/6J mice + LPS (intraperitoneal injection)	10 mg/kg (intraperitoneal injection)	-	↓ endotoxic shock and sepsis	
Guaianolides	Dehydrocostuslactone (6)	In vitro	THP-1 human cells + IL-6	6–25 μM	↓ IL-6/STAT3 and JAKs	↓ MCP-1, CXCL10, ICAM-1; ↓ STAT3 phosphorylation and DNA-binding activity; ↓ Intracellular GSH	[40]
		In vitro	Human keratinocytes from healthy donors + IL-22, IFN-γ or TNF-α	12.5 μM	↓ STAT3 and STAT1	↓ Intracellular GSH; ↓ CCL2, CXCL10, ICAM-1 and SOCS3; ↑ EGFR and Erk1/2	[41]
		In vivo	Colitis model: BALB/c mice + Dextran sulfate sodium (DSS) (oral administration)	10–20 mg/kg	↓ IL-6/STAT3	↓ TNF-α, IL-1β, MPO, SOD, IL-6, IL-17, IL-23, COX-2, iNOS	[50]
		In vitro	RAW 264.7 macrophages + LPS	10–20 μM	↓ MyD88/TRIF; ↓ NF-κB; ↓ IRF-3	↓ COX-2, INF-β, IP-10	[51]
	Micheliolide (7)	In vitro	BV2 microglia cells + LPS	1–10 μM	↓ NF-κB; ↓ PI3K/Akt ↓ MAPKs	↓ TNF-α, IL-6, IL-1β, COX-2, iNOS	[52]
		In vitro	RAW 264.7 macrophages + LPS	1–10 μM	↓ NF-κB	↓ IL-6, TNF-α, IL-1β	[53]
		In vitro	RAW264.7 macrophages + LPS	0–10 μM	↓ NF-κB; ↓ PI3K/Akt	↓ IL-6, TNF-α, MCP-1, INF-β and IL-1β	[54]
			Human dendritic cells and monocytes + LPS			↓ IL-6, TNF-α, MCP-1, INF-β	
		In vivo	Arthritis model: DBA/1 mice + collagen (intradermal injection)	30 mg/kg (intraperitoneal injection)	-	↓ TIMP-1, M-CSF, ICAM-1, INF-γ	[55]
	Cynaropicrin (8)	In vitro	RAW 264.7 macrophages + LPS	0–35 μM	-	↓ TNF-α and NO	[56]
			Human macrophages U937 + LPS				
			Primary splenocytes from mice + concanavalin A, phytohemagglutinin and LPS			↓ lymphocyte proliferation	
	Arglabin (9)	In vitro	Peritoneal macrophages from ApoE_2_.Ki mice + LPS and cholesterol crystals	50 nM	↓ NF-κB; ↓ NLRP3	↓ IL-1α, IL-1β, IL-18	[57]
	11β,13-dihydrolactucin (10)	In vitro	Yeast *S. cerevisiae* + MnCl_2_	0.36–18 μM	↓ Calcineurin-Crz1 (NFAT)	↓ NFAT nuclear translocation and transcriptional activity	[58]
	8-deoxylactucin (11)	In vitro	Human colon-cancer cells HT29 + TNF-α	115 μM	↓ NF-κB	↓ PGE_2_	[59]
Eudesmanolides	Alantolactone (12)	In vitro	bEnd.3 mouse endothelial cells + TNF-α	2.6–10.3 μM	↓ NF-κB and MAPKs	↓ IκBα, NF-κB p65, p38 and JNK phosphorylation	[26]
			RAW264.7 murine macrophages + LPS;			↓ IL-1, IL-6 and iNOS	
			Primary synovial fibroblasts from rheumatoid arthritis patients + TNF-α			↓ IL-1, MCP-1 and MMP-3	
		In vitro	RAW 264.7 macrophages + LPS	1.25–10 μM	↓ NF-κB; ↓ MyD88	↓ iNOS, COX-2, TNF-α	[60]
		In vivo	Colitis model: C57BL/6 mice + DSS (oral administration)	50 mg/kg (oral administration)	↓ NF-κB; ↑ hPXR	↓ iNOS, ICAM-1, COX-2, TNF-α, IFN-γ, IL-6	[61]
	Isoalantolactone (13)	In vitro	bEnd.3 mouse endothelial cells + TNF-α	2.6–10.3 μM	↓ NF-κB and MAPKs	↓ IκBα, NF-κB p65, p38 and JNK phosphorylation	[26]
			RAW264.7 murine macrophages + LPS			↓ IL-1, IL-6 and iNOS	
			Primary synovial fibroblasts from rheumatoid arthritis patients + TNF-α			↓ IL-1, MCP-1 and MMP-3	
		In vitro	293T cells + TNF-α	2.5–10 μM	↓ NF-κB and MAPKs	↓ UbcH5	[62]
		In vivo	Hepatitis model: BALB/c mice + TNF-α and D-galactosamine (D-GalN) (intraperitoneal injection)	10 mg/kg (intraperitoneal injection)		↓ serum alanine aminotransferase (ALT); ↓ hepatocyte damage; ↓ IL-6, MCP-1, ICAM-1 and VCAM-1	
	JEUD-38 (14)	In vitro	RAW 264.7 macrophages + LPS	2.5–10 μM	↓ NF-κB and MAPKs	↓ iNOS	[63]
	7-hydroxyfrullanolide (16)	In vitro	THP-1 cell + LPS	0.3–100 μM	↓ NF-κB	↓ NF-κB activation and nuclear translocation	[64]
			HUVECs + LPS			↓ ICAM-1, VCAM-1, E-selectin ↓ Monocyte adhesion	
			PBMCs + LPS			↓ NF-κB-related gene expression	
		In vitro	PBMCs + LPS	0.3–100 μM	-	↓ IL-6 and TNF-α	[65]
			Primary human synovial tissue cells				
		In vivo	Colitis model: BALB/c mice + DSS (oral administration)	75 mg/kg (oral administration)	-	↓ TNF-α and IL-6; ↓ Colonic edema; ↓ Shortening of the colon; ↓ hemoglobin and rectal bleeding; ↓ neutrophil infiltration	
			Paw edema model: Wistar rats + carrageenan (subcutaneous injection)	100 mg/kg	-	↓ paw edema	
			Arthritis model: DBA/1J mice + collagen (intradermal injection)	25–75 mg/kg (oral administration)	-	↓ joint deformities and bone destruction	
	Santamarin (17)	In vitro	RAW264.7 macrophages + LPS	5–40 μM	↓ NF-κB; ↑ Nfr2	↓ COX-2 and iNOS; ↓ TNF-α, IL-1β ↑ HO-1	[66]
			Murine peritoneal macrophages + LPS			↓ COX-2 and iNOS; ↓ TNF-α, IL-1β	
Heliangolides	Lychnopholide (18)	In vitro	J774A.1 macrophages + INF-γ and LPS	0.0125–0.2 μM	-	↑ IL-10; ↓ NO	[67]
	Eremantholide (19)		J774A.1 macrophages + INF-γ and LPS	0.625–10 μM	-	↑ IL-10; ↓ TNF-α	
	Budlein A (20)	In vitro	RAW264.7 + TNF-α or IL-1β	2.7 × 10^4^–26.7 μM	↓ NF-κB	↓ NF-κB activity	[68]
		In vivo	Arthritis model: C57BL/6 mice + methylated bovine serum albumin (intra-articular injection)	1–10 mg/kg (oral administration)		↓ edema; ↓ neutrophil and leukocyte infiltration; ↓ proteoglycan degradation; ↓ IL-33, TNF-α, IL-1β, COX-2	
		In vivo	Paw edema model: Swiss mice + carrageenan (subcutaneous injection)	1–10 mg/kg	-	↓ TNF-α, IL-1β; ↓ edema, and neutrophil infiltration; ↓ mechanical hypernocecipetion	[69]
		In vivo	Gout arthritis model: Swiss mice + monosodium urate crystals (intra-articular injection)	1–10 mg/kg (oral administration)	↓ NF-κB; ↓ NLRP3 inflammasome	↓ TNF-α and IL-1β; ↓ neutrophil recruitment; ↓ edema and mechanical hypersensitivity	[70]
		In vitro	Bone marrow derived macrophages (BMDMs) + LPS and monosodium urate crystals	2.7–26.7 mM		↓ TNF-α and IL-1β	
	Diacethylpiptocarphol (21)	In vivo	Colitis model: BALB/c mice + DSS (oral administration)	5 mg/kg (oral administration)	-	↓ TNF-α; ↑ TGF-β; ↓ immune cell infiltration and tissue damage	[71]
Pseudoguaianolides	Helenalin (22)	In vitro	Jurkat T cells + TNF-α	5–200 μM	↓ NF-κB	↓ NF-κB DNA-binding	[72]
		In vitro	Jurkat T cells + TNF-α	10 μM	↓ NF-κB	↓ NF-κB DNA-binding and nuclear translocation	[73]
		In vitro	Jurkat CD4^+^ T-cells	0.5–5 μM	↓ NFAT ↓ NF-κB	↓ IL-2 ↓ proliferation of CD4^+^ cells	[23,74]
		In vitro	THP-1 cells + LPS	0.52–1.08 μM	↓ NF-κB	↓ IL-1α, IL-19, MCP-3, GM-CSF	[75]
		In vitro	A2780 human ovarian cancer cell line	0.5–2 μM	↓ NF-κB	↓ NF-κB p65 expression	[76]
	11α,13-dihydrohelenalin (23)	In vitro	PBMCs + LPS	2–20 μM	↓ NF-κB and NFAT	↓ IL-2, IL-6, GM-CSF, TNF-α, INF-γ, iNOS	[77]
			Jurkat T-cells + LPS			↓ NF-κB and NFAT levels	
	11α,13-dihydrohelenalin–acetate (24)	In vitro	PBMCs + LPS	2–20 μM	↓ NF-κB and NFAT	↓ IL-2, IL-6, GM-CSF, TNF-α, INF-γ, iNOS	
			Jurkat T-cells + LPS			↓ NF-κB and NFAT levels	
		In vitro	Human granulocytes + Ionophore A23187	1–600 μM	↓ Arachidonic Acid	↓ Leukotriene C_4_ synthase; ↓ 5-lipooxygenase	[78]
Endoperoxide SL	Artemisinin (25)	In vitro	HUVECs + TNF-α	50–200 μM	↓ NF-κB; ↓ MAPKs	↓ ICAM-1, VCAM-1; ↓ adhesion of monocytes	[80]
	Dihydroartemisinin (26)	In vitro	RAW 264.7 macrophages + PMA	5–25 μM	↓ NF-κB, AP-1 and MAPKs	↓ COX-2	[81]

## Abbreviations

AA	Arachidonic acid
ADME	Absorption, Distribution, Metabolism, Excretion
Akt	Protein kinase B
ALT	Alanine aminotransferase
AP-1	Activator protein-1
apoE	Apolipoprotein E
BBB	Blood–brain barrier
BMDMs	Bone marrow-derived macrophages
CCL	Chemokine (C-C motif) ligand
CD	Cluster of differentiation
C/EBP	CCAAT-enhancer-binding protein
COX	Cyclooxygenase
Crz1	Calcineurin-responsive zinc finger 1
CSF	Colony-stimulating factor
CXCL10	C-X-C motif chemokine ligand-10
D-GalN	D-galactosamine
DMAPP	Dimethylallyl-diphosphate
DNA	Deoxyribonucleic acid
DSS	Dextran sulfate sodium
EGFR	Epidermal growth factor receptor
Erk	Extracellular signal-regulated kinase
FK506	Tacrolimus
FPP	Farnesyl diphosphate
GLUT	Glucose transporter
GM-CSF	Granulocyte-macrophage colony-stimulating factor
GSH	Glutathione
^1^H-NMR	Proton Nuclear Magnetic Resonance
HEK	Human embryonic kidney
HMGB	High mobility group box
HO-1	Heme oxygenase-1
hPXR	Human pregnane X receptor
HUVECs	Human umbilical vein endothelial cells
IBD	Inflammatory bowel diseases
IC_50_	Half maximal inhibitory concentration
ICAM	Intercellular adhesion molecule
IFN	Interferon
IκBα	NF-κB inhibitor alpha
IKK	IκB kinase
IL	Interleukin
iNOS	Inducible nitric oxide synthase
IP-10	Interferon gamma-induced protein-10
IPP	Isopentenyl diphosphate
IRF	Interferon regulatory factor
JAKs	Janus kinases
JNK	c-Jun N-terminal kinase
LDHA	Lactate dehydrogenase A
LOX	Lipoxygenase
LPS	Lipopolysaccharide
MAPKs	Mitogen-activated protein kinases
MCP	Monocyte chemoattractant protein
M-CSF	Macrophage colony-stimulating factor
MMP	Matrix metalloproteinase
MPO	Myeloperoxidase
mRNA	Messenger ribonucleic acid
MyD88	Myeloid differentiation primary response 88
NFAT	Nuclear factor of activated T-cells
NF-κB	Nuclear factor kappa-light-chain-enhancer of activated B cells
NLRP3	Nucleotide-binding oligomeriztion domain (NOD)-like receptor family pyrin domain-containing 3
NO	Nitric oxide
Nrf	Nuclear respiratory factor
NSAIDs	Nonsteroidal anti-inflammatory drugs
Oct-1	Octamer transcription factor-1
p38	Member of the MAPKs
p65	Subunit of NF-κB
PBMCs	Peripheral blood mononuclear cells
PDK	Phosphoinositide-dependent kinase
PGE_2_	Prostaglandin E_2_
PI3K	Phosphatidyl inositol 3-kinase
PKM2	Pyruvate kinase M2
PMA	Phorbol 12-myristate 13-acetate
PMNLs	Polymorphonuclear leukocytes
ROS	Reactive oxygen species
SAR	Structure-activity relationship
15(S)-HETE	15-Hydroxyeicosatetraenoic acid
SLs	Sesquiterpene lactones
SOCS	Suppressor of cytokine signaling
SOD	Superoxide dismutase
SOX	SRY-box transcrition factor 9
STATs	Signal transducer and activator of transcription
TGF-β	Transforming growth factor beta
Th1/Th2	Type-1/2 helper T lymphocytes
TIMP	Tissue inhibitor of metalloproteinases
TIRAP	Toll/interleukin-1 receptor domain-containing adapter protein
TLR	Toll-like receptor
TNBS	Trinitrobenzene Sulfonic Acid
TNF-α	Tumor necrosis factor alpha
TNFR	Tumor necrosis factor receptor
TPA	12-O-tetradecanoylphorbol acetate
TRAF	TNFR-associated factor
Trib1	Tribbles pseudokinase 1
TRIF	Toll-interleukin-1 receptor domain-containing adapter-inducing interferon-β
UbcH5	Ubiquitin-conjugating enzyme H5
VCAM	Vascular cell adhesion molecule
VEGF	Vascular endothelial growth factor
